# The future of collaborative precision oncology approaches in sub-Saharan Africa: learnings from around the globe

**DOI:** 10.3389/fonc.2024.1426558

**Published:** 2024-06-21

**Authors:** Amadou Gueye, Boutros Maroun, Amol Zimur, Tom Berkovits, Shen Mynn Tan

**Affiliations:** Illumina Inc, San Diego, CA, United States

**Keywords:** sub-Saharan Africa, research consortia, genomic diversity, cancer genomics, precision oncology, comprehensive genomic profiling

## Abstract

As the projected incidence and mortality of cancer in Sub-Saharan Africa (SSA) rises to epidemic proportions, it is imperative that more is done to identify the genomic differences and commonalities between patients of African and European ancestry to fulfil the promise of precision oncology. Here, we summarize the utility of precision oncology approaches, with a focus on comprehensive genomic profiling (CGP) and consolidate examples of national and international consortia that are driving the field forward. We describe the importance of genomic diversity and its relevance in cancer, and propose recommendations, success factors and desired outcomes for precision oncology consortia to adopt in SSA. Through this, we hope to catalyze the initiation of such projects and to contribute to improving cancer patient outcomes in the region.

## Introduction

Cancer continues to be a significant public health concern across sub-Saharan Africa, impacting millions within the region and contributing substantially to premature mortality in individuals aged 30 to 69 years (1). Notably, cancers such as breast, cervical, prostate, and colorectal cancer predominate, reflecting a complex interplay of genetic, environmental, and lifestyle factors. Despite historical emphasis on communicable diseases like Malaria, Tuberculosis, and HIV/AIDS, the rising incidence and mortality rates of cancer underscore the urgent need for comprehensive cancer control strategies in Africa (2). In 2020, there were 1,185,216 new cancer cases in Africa, with prostate and breast cancers being the most prevalent in males and females respectively (3). Projections suggest there will be a nearly twofold increase in cancer incidence and mortality by 2040, primarily due to population growth, aging, and lifestyle changes (1).

Despite the looming crisis, financing for cancer prevention, diagnosis, and treatment remains inadequate in many SSA countries. The economic burden of cancer further strains healthcare systems, emphasizing the urgent need for increased investment and innovative funding models. The gaps in cancer surveillance and control in SSA are also due to inadequate data infrastructure to limited access to screening and treatment services (4). Compared to other regions, Africa suffers from an acute lack of diagnostic capacity, epidemiological data collection, training and research on specific risk factors or needs to tailor specific interventions to the most prevalent cancers in Africa. Even among African countries, significant disparities exist in cancer diagnosis and screening capabilities, contributing to variations in cancer incidence and mortality rates (5).

## Precision oncology

Precision oncology is rapidly reshaping cancer care by increasing utilization of genomic profiling for diagnosis and treatment guidance. In recent years, the FDA has approved numerous drugs across different solid tumors based on specific genomic targets or biomarkers, which can be attributed to advances in our understanding of cancer biology and the rapid development of high-throughput technologies (6). Thus, an in-depth understanding of the tumor’s genetic features and actionable alterations is crucial for selecting the most appropriate targeted therapies or immunotherapies, eligibility for clinical trials, and ultimately improving patient outcomes and survival rates (7, 8). This is especially important as cancer characterization is moving away from the traditional focus of where the tumor is in the body, to a molecular classification of tumors that is better able to guide targeted therapies (9).

By providing a holistic view of the genomic drivers of the hallmarks of cancers, genomic profiling can identify multiple cancer-related genes in a single assay as well as rare or novel mutations that may not be detected through conventional testing methods, thus guiding personalized treatment strategies tailored to the specific genetic profile of the individual patient (10). Together with the molecular characterization of tumors, the use of Molecular Tumor Boards (MTBs) can help achieve effective interpretation of multiple testing approaches leading to MTB-recommended therapy that is better matched to the patient’s genomic alterations than physician’s choice therapy (11).

## Comprehensive genomic profiling

The recent introduction of CGP enables a thorough analysis of the entire genomic landscape of a tumor sample via large panel Next-Generation Sequencing (NGS) testing. This approach goes beyond traditional assays: immunohistochemistry (IHC), polymerase chain reaction (PCR), fluorescence *in situ* hybridization (FISH), single-gene and small panel testing. It offers a more comprehensive assessment of genetic alterations on both the DNA and RNA levels that occur across all tumor types (12), including single nucleotide variants (SNV), insertions or deletions (indels), copy number variations (CNV), fusions, and rearrangements across the entire genome. It can also detect more actionable biomarkers compared to conventional testing methods, for example in melanoma where ~37% more patients with *BRAF* alterations were identified with CGP compared to other techniques (13). In addition, CGP can accurately measure genomic signatures such as tumor mutational burden (TMB), microsatellite instability (MSI), and homologous recombination deficiency (HRD) (14–16).

CGP thus allows for a more comprehensive analysis of a patient’s tumor genome, identifying genetic mutations and alterations that drive cancer progression (17). This has enabled oncologists to tailor treatment plans based on the unique genetic profile of the patient, leading to improved therapeutic outcomes and personalized care (14, 18, 19). Thus, both American Society of Clinical Oncology (ASCO) and European Society for Medical Oncology (ESMO) guidelines recommend large NGS panel testing in patients with advanced cancers for accurate biomarker profiling (20, 21). As the landscape of cancer treatment continues to evolve, CGP holds immense promise for guiding clinical decision-making, optimizing treatment strategies, and ultimately improving patient outcomes in the era of precision oncology.

## National and international implementation

The implementation of precision oncology requires building upon its utility at a systems level where complex, multi-layered datasets are transformed into clinically useful information that guides patient management whilst including real-world evidence-based (RWE) studies that can expand the spectrum of therapeutic options (22). Many such national-level precision oncology programs have been initiated in the last decade, with countries in Europe leading the way; both “top-down” national strategies and infrastructure investment and “bottom-up” regional network competency development have been employed (23). One recent example of successful implementation is the UK National Healthcare System (NHS), which published their integrated genomic and real-world data (RWD) from the Cancer Programme of the 100,000 genomes project describing the utility of whole genome sequencing (WGS) in close to 14,000 solid tumors spanning more than 30 cancer types (24). In a departure from the more common government-funded model, a more sustainable approach is being trialed in Australia, where Omico (a non-profit organization) has been established with both public and private funding to spearhead their national precision oncology strategy by integrating CGP-informed clinical trials into the standard of care (25).

With the increase in the amount of clinical data generated for each patient (both traditional clinical variables and new omics data), the challenges of consolidating, understanding, and utilizing this to benefit patients cannot be understated. Continuous monitoring of such RWD with their Clinical Communication Platform (CCP) allows the German Cancer Consortium (DKTK) to leverage comprehensive data from diagnostics to treatment response of over 200,000 patients to monitor clinical pathways and outcomes (26). This can then be used to inform future clinical trial design and can be a catalyst for translational research. Such platforms can also push the boundaries of both cross-border cooperation and potential patient benefits. One example is the LANTERN project, a multi-center observational clinical trial involving five institutions from different European countries, which seeks to employ advanced machine learning (ML) and artificial intelligence (AI) to develop predictive models for lung cancer diagnosis and predictive individual-specific treatments (27). This leveraging of RWD and RWE can not only guide treatment decisions but also support regulatory decision-making by national and EU-wide bodies (28).

Additional benefits demonstrated by international consortia include the ability to power research questions with sufficiently large cohorts and the establishment of consensus regional guidelines. The Pancreatic disease research consortium (PANDoRA), 29 groups across 12 European countries including Brazil and Japan, was able to contribute to the discovery of 25 susceptibility loci in ten years (29); whilst the Asia Pacific Oncology Drug Development Consortium (APODDC) recently published its recommendations for the use of next-generation sequencing (NGS) in patients with metastatic cancer in the Asia-Pacific region (30).

## Genomic relevance

Given the importance of cancer genomics in precision oncology, a key factor for success will be the relevance of consortium-generated data to the population in question. It has been found that minorities do not have sufficient representation in large international cancer registries like the Project Genomics Evidence Neoplasia Information Exchange (GENIE) (31). Indeed, cancer incidence and survival differ by race in US registries, with individuals of African ancestry having a globally increased risk of malignancies compared with their Asian and Caucasian counterparts (32).

Differences in genomics, incidence and mortality across race and ethnicity have been described for a range of cancers, such as prostate cancer (33), early onset colorectal cancer (34), breast invasive carcinoma and head and neck squamous cell carcinoma (35). In advanced non-small-cell lung cancer, average TMB was shown to be highest in patients of African ancestry and lowest in patients of Asian ancestry, potentially impacting the stratification of patients who would be eligible for immunotherapy (36). Thus, getting the right drug to the right patient requires the right genomics, especially for tumor agnostic treatments, will potentially lead to improved survival outcomes (37).

## Challenges in Africa

Addressing challenges of the cancer burden in SSA demands innovative and context-specific approaches. It is critical to build robust cancer surveillance and reporting systems, data infrastructure, and foster cross-sectoral collaboration. In addition to surveillance, innovative funding strategies are needed to expand access to screening, diagnosis, and treatment services. Mobile health technologies, community-based outreach programs, and task redistribution approaches will help overcome barriers to care and reaching underserved populations (2).

Cancer genomics holds immense promise for transforming cancer care in SSA. By elucidating the genetic drivers of cancer and identifying biomarkers for early detection and targeted therapies, genomics can enable personalized treatment approaches and potentially improve clinical outcomes. Challenges such as limited infrastructure, human resource capacity and cost constraints hinder the widespread adoption of genomic technologies in SSA. Collaborative efforts involving governments, academia, industry, and international organizations are needed to overcome these barriers and harness the full potential of cancer genomics in the region (38).

## Suggestions for consortia

**Figure 1:**
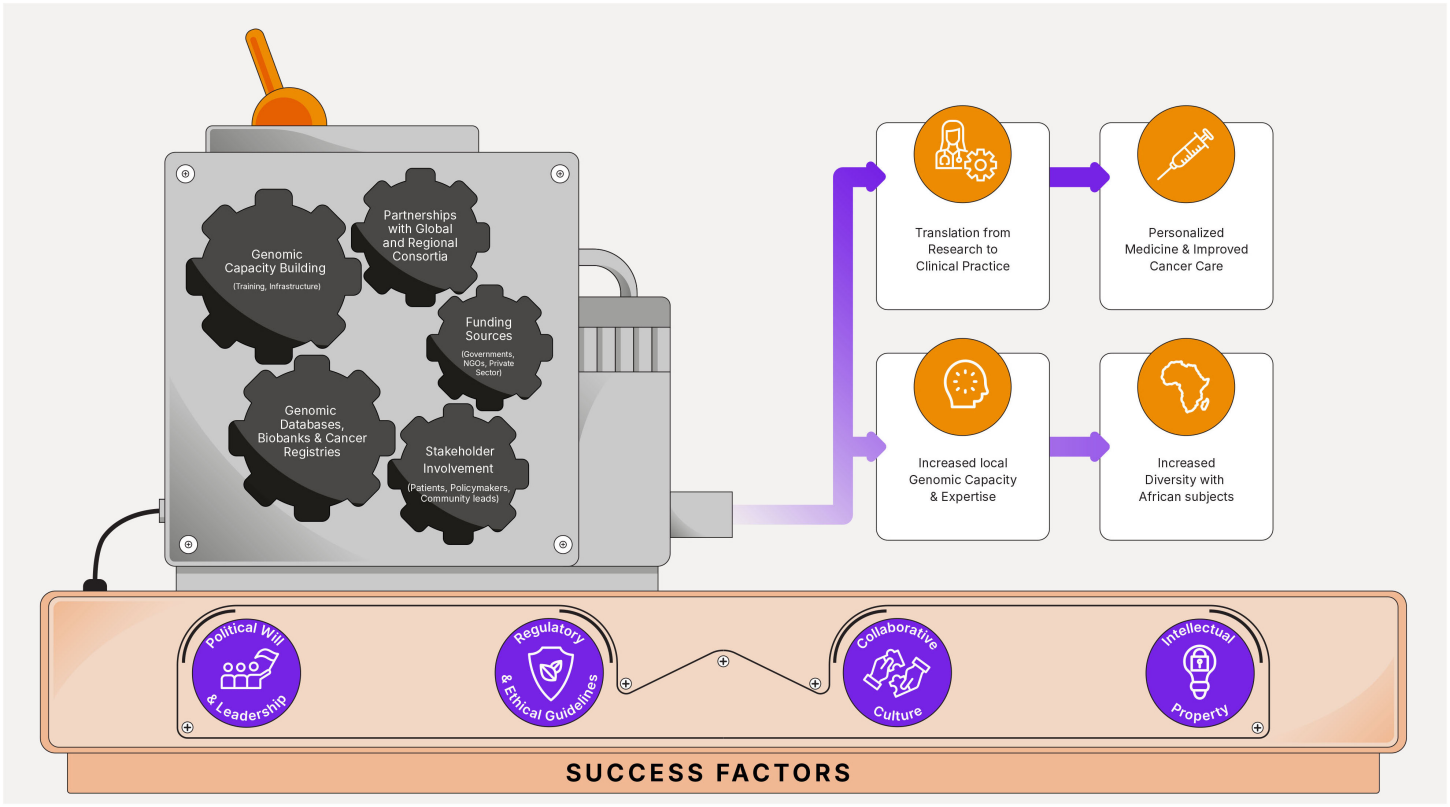
Precision oncology consortia in Sub-Saharan Africa: success factors, recommendations, and potential outcomes.

Adopting a consortia approach in cancer genomics can be highly beneficial for SSA, where resources and expertise may be limited. By pooling resources, expertise, and data, consortia can accelerate research, improve understanding of cancer genetics, and facilitate the development of and access to targeted therapies tailored to populations in the region (39). Here we share some recommendations for consortia approaches in cancer genomics specific to SSA (**Figure 1**).

### Collaboration with existing global consortia

Partner with global initiatives such as the International Agency for Research on Cancer (IARC), the International Cancer Genome Consortium (ICGC), the Human Heredity and Health in Africa (H3Africa) project and the African-Caribbean Cancer Consortium (AC3) to leverage existing resources, expertise, network and infrastructure for cancer genomics research.

### Establishment of regional consortia

Work with regional consortia that bring together researchers, clinicians and policymakers from multiple countries within Sub-Saharan Africa. This can foster collaboration, resource sharing, and the exchange of knowledge and best practices. The following groups are already very active in this field and are a good place to start: the African Organization for Research and Training in Cancer (AORTIC), the African Consortium for Cancer Clinical Trials (AC3T), the Consortium for Head and Neck Cancer in Africa (ChancAfrica), The African Esophageal Cancer Consortium (AfrECC), The Africa HepatoPancreatoBiliary Cancer Consortium (AHPBCC) (40).

### Securing funding

Seek funding from a variety of sources including government agencies, international organizations, philanthropic foundations, and private sector partners to support consortium activities, infrastructure development, and research projects. More and more African governments are allocating domestic funding for cancer research and care but that is not enough to cover all needs. Therefore, involving philanthropic foundations and NGOs that are supporting the fight against cancer and forging partnerships with private companies in pharmaceuticals, biotechnology or healthcare will be key for success.

Successful models of public-private partnerships in funding cancer centers and increasing access to new diagnostic technologies exist in several African countries and should be replicated (2, 40).

### Capacity building initiatives

Develop capacity building initiatives to train researchers and healthcare professionals in cancer genomics technologies, bioinformatics, and data analysis. This will help build local expertise and empower African researchers to conduct genomics research in their respective countries. The African Organization for Research and Training in Cancer (AORTIC) and the Union for International Cancer Control (UICC) are the main groups leading the effort in building capacity in Africa.

### Data sharing platforms

Establish platforms for researchers across SSA to access and analyze genomic data collected from studies and cancer registries. These platforms should adhere to ethical guidelines and data protection regulations while promoting open science principles. The African Cancer Registry Network (AFCRN), the H3Africa Bioinformatics Network (H3ABioNet) and the Data Science for Health Discovery and Innovation in Africa project (DS-I Africa) are good examples of data sharing platforms.

### Incorporate diversity in genomic studies

Ensuring more diversity in genomic studies by including samples from diverse populations within SSA is crucial. This will help identify population-specific genetic variations associated with cancer susceptibility, progression, and treatment response (41).

### Engagement with stakeholders

Involve stakeholders such as patient advocacy groups, community leaders, and policymakers in consortium activities to ensure that research priorities align with the needs and concerns of local communities.

### Translation of research findings

Facilitate the translation of research findings into clinical practice by working closely with healthcare providers, regulatory authorities, and industry partners to develop and implement targeted therapies and personalized treatment approaches for cancer patients in Sub-Saharan Africa (41).

## Conclusion

Given the increasing incidence and mortality of cancer in SSA, it is imperative that all stakeholders work together to address this issue. Leveraging work already done and ongoing by consortia around the globe, both public and private institutions can and should come together to solve key issues and improve both knowledge and expertise in Africa. We hope that the suggestions contained herein will spur increased discussion and further collaboration in precision oncology in the region, thus leading to improved cancer patient outcomes in SSA in the near future.

## Data availability statement

The original contributions presented in the study are included in the article/supplementary material. Further inquiries can be directed to the corresponding author.

## Author contributions

AG: Conceptualization, Investigation, Writing – original draft, Writing – review & editing. BM: Conceptualization, Investigation, Writing – original draft, Writing – review & editing. AZ: Writing – review & editing. TB: Writing – review & editing. ST: Conceptualization, Investigation, Supervision, Writing – original draft, Writing – review & editing.
